# Agency Attribution in Infancy: Evidence for a Negativity Bias

**DOI:** 10.1371/journal.pone.0096112

**Published:** 2014-05-06

**Authors:** J. Kiley Hamlin, Andrew S. Baron

**Affiliations:** Departent of Psychology, The University of British Columbia, Vancouver, Canada; Boston College, United States of America

## Abstract

Adults tend to attribute agency and intention to the causes of negative outcomes, even if those causes are obviously mechanical. Is this over-attribution of negative agency the result of years of practice with attributing agency to actual conspecifics, or is it a foundational aspect of our agency-detection system, present in the first year of life? Here we present two experiments with 6-month-old infants, in which they attribute agency to a mechanical claw that causes a bad outcome, but not to a claw that causes a good outcome. Control experiments suggest that the attribution stems directly from the negativity of the outcome, rather than from physical cues present in the stimuli. Together, these results provide evidence for striking developmental continuity in the attribution of agency to the causes of negative outcomes.

## Introduction

Animate agents are capable of goal-directed action and inanimate objects are not. The capacity to distinguish these two kinds of entities is essential to human survival: recognizing the tube-like green object in the grass as a snake and not a hose could save us from a deadly bite. In addition to adaptively constraining approach and avoidance, representations of agents and their mental states guide important social behaviors such as whom to learn from (e.g., distinguishing knowledgeable sources from ignorant ones), whom to hold morally and legally responsible (e.g., distinguishing intentional from accidental harm), and underlies the capacity for uniquely human social-emotional cognitions (e.g., deception; humor). Underscoring the critical nature of accurate agency detection, a failure to automatically perceive and/or to reason about agents may underlie broad deficits in social functioning such as autism-spectrum disorders [1], [2], [3].

Notably, it is seemingly always better to over-attribute agency than to under-attribute it [4], [5]. For instance, whereas mistaking one's hose for a snake could lead to the death of one's lawn, mistaking a snake for one's hose could lead to the death of one's self: arguably a far more negative outcome. Perhaps due to this cost differential, typically-developing adults tend to *over-attribute* agency to entities in the world, regularly ascribing perceptions, intentions, and beliefs to mechanistic objects like computers, to meteorological events like tornadoes, and to random acts of chance like winning the lottery [6]–[13]. This global tendency to attribute agency to non-agents appears to have a parallel in how actual agentive actions are processed: adults display enhanced memory for individuals who helped or hindered a third party intentionally versus accidentally [14]. and are biased to view even explicitly accidental human actions as goal-directed and intentional unless given the time and motivation to do otherwise [15].

Both the essential nature of agency detection and the ubiquity of agency over-detection has inspired what is now a very large body of research into when and how agency representations develop, including how agents are identified and how mental state reasoning is applied to their actions [16]–[38]. Sharp theoretical differences exist amongst various developmental accounts, in particular with respect to whether agency representations are seen as the result of accumulated experience with actual agents in the world including the self [27], [28], [36]. or are built on “pre-wired” agency attribution systems that are sensitive to various cues to agency [17], [24], [26], [39]. These theoretical differences aside (see also [34]), this research has identified several classes of characteristics that reliably inspire agency attribution in infancy. First, infants attribute agency to things that *look like* agents: that have eyes, a face, or a body. Second, infants attribute agency to things that *move like* agents: that are self-propelled and that exhibit non-inertial patterns of motion. Third, infants attribute agency to things that *act like* agents: that approach end-states efficiently, approach the same end-state from multiple angles, and that vary their motion based on changes in the physical environment; all of which imply that a given action is goal-directed. Finally, infants attribute agency with things that *interact like* agents, for example, that effect a physical change in the environment or respond in a contingent, turn-taking manner.

Interestingly, one of the most well studied cues to agency in adulthood has been relatively absent from infancy research: the valence of an action's effect ([6]–[9], [39]–[41], see [12], [42] for research with children). That is, adults are especially likely to infer that an agent was the cause of particularly positive or particularly negative outcomes; in particular, negative outcomes seem to be relatively stronger cues to agency than are positive outcomes. For example, while it is difficult to imagine praising a computer that is functioning well, adults spontaneously scold a computer that fails to meet their needs [43] and attribute more agency to computers that malfunction more often [44]. In addition, when asked to guess whether a game outcome originated from a computer or a human agent, adults attribute negative outcomes to an external agent but attribute both neutral and positive outcomes to random chance, even when they know that all outcomes are equally likely [41]. This phenomenon, which Moorewedge [41] has recently dubbed the “negative agency bias,” may also account for adults' tendencies to ascribe more intentionality to negative than to positive side-effects of planful agentive actions (even if all side-effects are explicitly marked as unintended; [39], [40]), and to attribute agency to decidedly inanimate objects (robots and dead people) that have been targeted by acts that typically bring about negative outcomes (assault; [45]).

Given the amount of research devoted both to agency attribution in infancy and to the negative agency bias in adulthood, it is fairly surprising that there has been little exploration of whether infants' agency representations are sensitive to valence. That said, there is evidence from various developmental paradigms that infants, like adults, may show a more general “negativity bias,” by which negative elements in the environment are given more attention, memory, and causal reasoning resources than are positive or neutral ones (see [46] for a review of the developmental work; for reviews of adult work see [47], [48], [49]), and several recent developmental studies have demonstrated that this bias with regards to negative social information in infancy and early childhood. For example, young children show relatively better memory for mean than for nice individuals [50], infants more readily adjust their approach behaviors toward novel objects/situations when given negative rather than positive information from their caregivers (reviewed in [46]), older infants selectively avoid following preference information provided by antisocial others but treat prosocial and unknown others as equally good sources of information [51], and young infants negatively evaluate those who hinder others' goals before they positively evaluate those who facilitate others' goals [52]. Despite this work, no previous work has examined specifically whether infants use negative (or positive) valence as a cue to agency.

There are both theoretical and methodological reasons for this lack of research into the role of outcome valence and agency representations in infancy. First, despite the fact that researchers have discussed the relative survival benefits agency over-attribution may have provided our more agent-sensitive ancestors [4], [6], [9], [53], negatively-valenced agency biases in particular are often attributed to various forms of motivated reasoning, allowing individuals to avoid blame and uncertainty surrounding negative outcomes [54]–[59]. These self-protective processes presumably require a conscious sense of self and an explicit desire to save “face”; infants may lack these capacities. In addition, the lack of developmental research may stem from the fact that “valence” is a fairly ambiguous term, and it may have been unclear how to operationalize it in infancy. To illustrate this difficulty, adults' valenced agency representations have been studied using good/bad outcomes experienced by oneself (wins or losses in a game; [41]), good/bad outcomes experienced by others (positive or negative side effects from some fictitious program; [39]), and actions that *typically* lead to a good/bad outcome, but just do not do so in this case, such as assault that does not lead to harm because the victim is a robot [60]. Finally, with some notable exceptions [20], [61]. until recently there has been relatively little research into whether infants attribute positive or negative valence to particular actions, outcomes, or intentions at all; thus, operationalizing valence for the purpose of exploring the development of valenced agency biases in infancy may have been difficult (but see [62] for work with children). Despite these difficulties, a more complete understanding of the foundations of agency detection in infancy, in particular one that considers the role of valenced outcomes in infants' tendency to attribute agency to entities in the world, would speak both to the true nature of adults' agency representation system as well as to the richness of infants' earliest representations of agents. This is the aim of the current studies.

## The current studies

The current studies ask whether infants, like adults, are biased to attribute agency to entities that have brought about valenced outcomes. Recent research suggests that infants prefer those who facilitate others' goals to those who block them by 3 to 6 months of age, suggesting that infants positively evaluate helping and/or negatively evaluate hindering [52], [63], [64]. These evaluations presumably require that infants have assigned positive valence to goal achievement and negative valence to goal failure. Here we explore whether 6-month-old infants attribute agency to *a mechanical claw* that previously either facilitated (Opener condition) or blocked (Closer condition) an agent from reaching its goal to open a box [63]. Crucially, previous work has shown that infants fail to attribute agency to a claw [26], [37], [65], [66], unless it exhibits specific cues to agency that are not present in the current stimuli [16]. We reasoned that if outcome valence (positive and/or negative) is a cue to agency in infancy, 6-month-olds should look longer to events in which a valenced claw “changes its mind,” or acts inconsistently with its previous goal-directed act, as they do when viewing acts performed by a human hand but not those performed by an unvalenced claw [37]. Alternatively, if valenced outcomes are not a cue to agency in infancy, 6-month-olds should not increase their attention to goal-change events. The Opener condition examines whether infants use positive outcomes as a cue to agency; the Closer condition examines whether infants use negative outcomes as a cue to agency.

## Experiment 1

### Methods

#### Ethics statement

This research was approved by the Behavioural Research and Ethics Board at the University of British Columbia; written informed consent was given by the parents or guardians of each participant.

#### Participants

Participants were 40 6-month-olds (21 males; mean = 6;2; range: 5;2–6;16) recruited from a metropolitan area. Twenty infants were randomly assigned to the Opener condition (8 females; range: 5;16–6;16) and 20 to the Closer condition (11 females; range: 5;16–6;15). An additional 19 infants were tested but excluded due to fussiness (5 in the Opener condition, 8 in the Closer condition; fussiness rate did not differ by condition: Fisher's Exact *p* = .54) experimental error (4 in the Opener condition, 1 in the Closer condition), and parental interference (1 in the Closer condition).

#### Disclosure on sampling procedure

In an original sample there were 16 infants per condition. During preliminary hypothesis testing a significant effect in one condition and a marginal interaction between conditions were found; 4 additional infants were subsequently added to each condition of Experiment 1; adding 25% more subjects per condition is fairly common in this statistical situation, given the difficulty with acquiring infant participants.

#### Materials and procedure

Infants sat on their parent's lap before a table with a curtain at the far end (111 cm from the infant) that could be lowered to occlude a puppet stage. Parents were instructed not to attempt to influence their infants in any way; in addition, parents closed their eyes during critical (test) trials.

#### Familiarization Events

**Figure 1:**
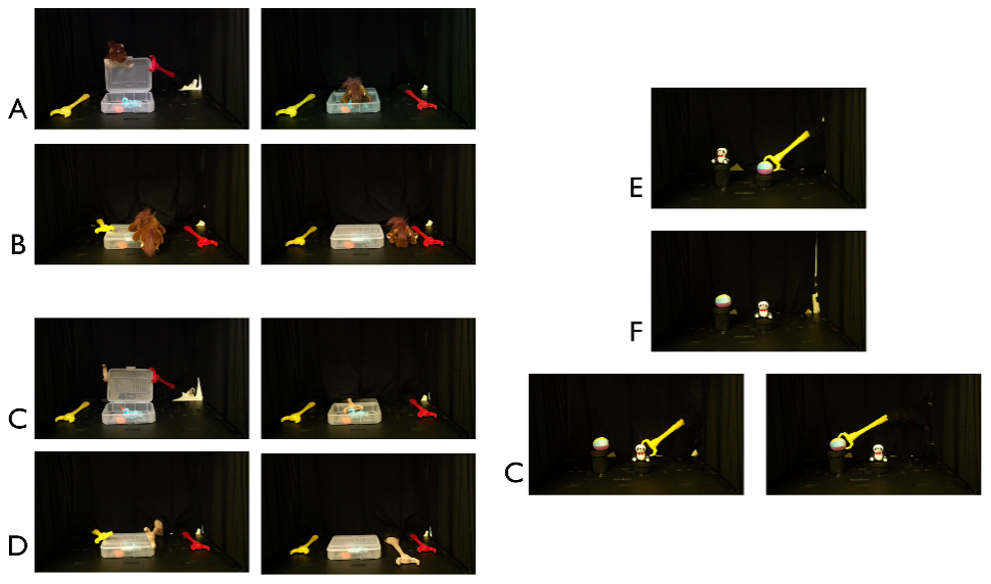
Stimuli. Panels A–B: Familiarization events for Experiment 1. A) Positive Outcome Condition: Protagonist enters and attempts to open box. Helpful Claw opens box with Protagonist. Protagonist grasps toy inside box; Helpful Claw returns to initial position next to box. B) Negative Outcome Condition: Protagonist enters and attempts to open box. Unhelpful Claw rises up and pushes box lid down. Protagonist puts head down next to box; Unhelpful Claw returns to initial position next to box. Panels C–D: Familiarization events for Experiment 2. C) Opener Condition: Brown Claw attempts to open box. Opener Claw opens box with Brown Claw. Brown Claw grasps toy inside box; Opener Claw returns to initial position next to box. D) Closer Condition: Brown Claw attempts to open box. Closer Claw rises up and pushes box lid down. Brown Claw puts head down next to box; Closer Claw returns to initial position next to box. Panel E: Habituation events. Claw from Familiarization enters from behind curtain on right of stage; grasps object. Panel F: Static Baseline Event. Toys have changed location from habituation. Panel G: Test events. During NewGoal events, Claw grasps new object in old location. During NewPath events, Claw grasps old toy in new location.

Depicted in Figure 1; actions are modeled from box events from Hamlin & Wynn [63]. A clear box with a toy inside rested onstage; a mechanical claw rested on each side of the box, one covered in red duct tape, the other in yellow. A puppeteer controlled the claws from behind a curtain; her hands were not visible. At the start of each event, a brown horse agent entered from behind a curtain at the back of the stage, moved to one side of the box, and appeared to “look” at the toy inside. The horse then grasped the lid and struggled unsuccessfully to open the box. On the horse's 5^th^ struggle, the claw resting on the opposite side of the box intervened. The color (red or yellow) and side (to the right or left of the box) of the intervening claw was counterbalanced within each condition.

In the Opener condition (Figure 1A), the Opener claw rose straight up from the stage floor, grabbed the opposite corner of the box lid, and opened the lid of the box together with the horse. The horse put his head down inside the box, achieving his goal to grasp the toy; this was a positive outcome for the horse. In the Closer condition (Figure 1B), the Closer claw rose straight up from the stage floor, moved straight over toward the box, and pushed straight down on the top of the box, shutting the lid. The horse put his head down next to the box, failing to achieve his goal to grasp the toy; this was a negative outcome for the horse. In both conditions, after the horse had lowered his head, the Opener/Closer claw returned to its original location on one side of the box, and all action paused. Infants' looking time was recorded online from this point by a coder peeking through a hole in the curtain utilizing the program *jHab* [70]. Total looking time was amassed until an infant looked away from the stage for 2 consecutive seconds, or until 30 seconds elapsed. The same familiarization event (Opener or Closer) was then repeated for a total of two events.

#### Habituation Events

Figure 1E. Infants in the Opener and Closer condition saw identical habituation events, which were modeled exactly after Woodward (1998). The curtain rose to reveal two toys (ball and bear; side counterbalanced) sitting atop two black pedestals, one tall (15.5 cm) on the (infant's) left and one short (8 cm) on the right, 11 cm apart. The Opener or Closer from familiarization (depending on the infant's condition) entered from behind the curtain on the infant's right and grasped either the toy on the right (near) pedestal or the toy on the left (far) pedestal (side counterbalanced). Action paused once the claw grasped the toy; infants' looking time was recorded from this point as during familiarization. Identical grasping events repeated until infants reached a pre-set habituation criterion indicating they had sufficiently processed the grasp; this criterion was met when the total attention to any three consecutive habituation events was less than half the total attention to the first three habituation events. Infants who did not meet the criterion were shown 14 total events.

#### Toy-Location-Switch Event

Figure 1E. The curtain rose to reveal the toys had switched locations, and rested on opposite pedestals. Infants' looking time to this static event was recorded from the point both toys were visible as previously.

#### Test Events

Figure 1G. Parents closed their eyes during test events. The toys remained in their new locations, atop the opposite pedestal from habituation. During each test event, the claw entered from behind the curtain on the infant's right and grasped each toy in alternation for a total of 6 test events. During *New Goal* events, the claw moved along the same path as in habituation toward the same pedestal, but grasped the toy that now rested there, which had not previously been grasped. During *New Path* events, the claw grasped the same toy as in habituation, but did so by moving along a new path toward the opposite pedestal. Looking time was recorded from the point the claw grasped a toy as previously; the order of New Goal/New Path events was counterbalanced in each condition. A second independent coder, blind to condition, re-coded a random 25% of subjects' test events; the two coders reached 98% agreement. Additionally, we calculated the difference score between the original coder and the independent coder on each trial and computed the number of times that difference was in the hypothesized direction. This occurred on 31 out of the 60 recoded test trials.

### Results

#### Attention to Familiarization/Habituation events

A repeated-measures ANOVA with attention to familiarization, attention to the first 3 habituation events, and attention to the last three habituation events as within-subjects factors and condition as a between-subjects factor revealed a significant effect of condition (F_2,76_ = 3.31, *p*<.05, η_p_^2^ = .08). Subsequent between-condition comparisons revealed that infants attended significantly longer following Closer than Opener familiarization events ((average of both) Closer = 8.13s (SEM = 1.25); Opener = 4.53s (SEM = .59); F_1,38_ = 6.74, *p*<.05; η_p_^2^ = .15), but that infants in the Closer condition did not subsequently attend significantly longer than those in the Opener condition to either the first 3 or the last 3 grasping habituation events (first3hab_Closer = 7.72 s (1.18), first3hab_Opener = 5.62 s (.71), F_1,38_ = 2.33, *p*>.13; η_p_^2^ = .06; last3hab_Closer = 3.45 s (.52), last3hab_Opener = 3.61 s (.87), F_1,38_ = .02, *p*>.87; η_p_^2^ = .00). Rate of habituation did not differ by condition: infants in the Closer condition habituated in an average of 9.6 events (SEM = .72; 4/20 did not habituate in 14 trials), and infants in the Opener condition habituated in an average of 9.9 events (SEM = .70; 5/20 did not habituate in 14 trials; univariate t_38_ = .27, *p*>.78, η^2^ = .002).

#### Attention to New Goal versus New Path test events: Preliminary analyses

There were no overall condition differences in attention during test; that is, the object-directed actions of a claw that previously caused a negative outcome were not on the whole more interesting to infants than were the object-directed actions of a claw that had previously caused a positive outcome (AverageTestAttention_Closer_ = 4.46 s (.39), AverageTestAttention_Opener_ = 4.10 s (.30), F_1,38_ = .28, *p*>.60, η_p_^2^ = .007). A preliminary repeated-measures ANOVA on infants' looking times to New Goal versus New Path test events with sex, whether or not the infant had habituated in 14 trials, claw color, claw side during familiarization, targeted toy (ball or bear), targeted toy side during habituation, and order of New Goal/New Path events during test as between-subjects factors, and with age, attention during familiarization, attention during the first 3 habituation trials, and attention during the last three habituation trials as covariates, revealed only a marginal effect of the side of the claw's grasps during habituation (F_1,4_ = 5.95, *p* = .07, η_p_^2^ = .60); there were no other marginal or significant effects (although this ANOVA had a large number of variables, grouping variables and performing several smaller repeated-measures ANOVAs yielded no additional effects). A follow-up repeated-measures ANOVA with targeted-toy-side as the single between-subjects variable revealed a significant effect (F_1,36_ = 6.85; *p*<.05; η_p_^2^ = .15): across both conditions infants who viewed the claw grasp the toy on the far pedestal during habituation were more likely to distinguish New Goal from New Path events than were those who saw the claw grasp the toy on the near pedestal during habituation. Although the reason for this influence of side on attention was unknown, as it significantly influenced infants' attention to New Goal versus New Path test events it was retained as a between-subjects variable in the analysis that follows; all other variables were collapsed for subsequent analyses.

#### Attention to New Goal versus New Path test events: Main analysis

To examine whether viewing a mechanical claw cause a positive and/or a negative outcome for an agent influences infants' tendency to attribute goal-directedness to that claw, we performed a repeated-measures ANOVA on infants' looking to New Goal versus New Path events, with both condition (Opener/Closer) and targeted-toy-side (right/left) as between-subjects variables. This analysis revealed no significant between- or within-subjects main effects (F's>.3), but there were significant interactions of infants' attention to New Goal versus New Path events with both condition (F_1,36_ = 6.20, *p*<.05, η_p_^2^ = .15) and targeted-toy-side (F_1,36_ = 7.79, *p*<.01, η_p_^2^ = .18). No 3-way interaction between trial type, condition, and side was observed (F_1,36_ = . 98; *p* = .33; η_p_^2^ = .03; this interaction of targeted-toy side with infants' attention to New Goal versus New Path events mirrored the results of the preliminary ANOVAs. As this effect did not differ by condition, and because an independent interaction with condition emerges when targeted-toy side is included as a between-subjects variable in the analysis, targeted-toy side was removed from further analyses in Experiment 1). The significant interaction between trial type and condition suggests that infants did not attribute goal-directedness to claws that acted on an agent's goal across the board; rather, infants' attributions differed depending on whether the claw had previously helped an agent – causing a positive outcome – or previously harmed an agent – causing a negative outcome.

Planned contrasts suggest that infants in the Closer condition treated the claw as an agent: they significantly dishabituated to events in which the claw grasped a new object (last3hab_Closer_ = 3.45 s (.52), NewGoalTest_Closer_ = 4.95 s (.58); paired t_19_ = −2.43, *p*<.05; η^2^ = .24) but not to events in which the claw grasped the same object via a new path of motion (last3hab_Closer_ = 3.45 s (.52), NewPathTest_Closer_ = 3.99 s (.61); paired t_19_ = −.91, *p*>.37; η^2^ = .04). In addition, infants in the Closer condition looked significantly longer to New Goal events than to New Path events (paired t_19_ = 2.18, *p*<.05; η^2^ = .20). In contrast, infants in the Opener condition showed no evidence of treating the claw as an agent: they failed to dishabituate to either New Goal or New Path events (last3hab_Opener_ = 3.61 s (.87), NewGoalTest_Opener_ = 3.91 s (.42), t_19_ = −.28, *p*>.77; η^2^ = .004; NewPathTest_Opener_ = 4.33 s (.51); paired t_19_ = −.76; *p*>.45; η^2^ = .03), and looked equally to New Goal and New Path events (paired t_19_ = −1.02, *p*>.31, η^2^ = .05). These patterns were reflected in individual infants' tendency to look longer to New Goal events than to New Path events during test: 16 of 20 infants in the Closer condition looked longer to New Goal than to New Path events (binomial *p*<.05), whereas only 9 of 20 infants in the Opener condition did so (binomial p>.82; Pearson's χ^2^ = 5.23, *p*<.05).

#### Is this effect due to attention during familiarization?

Although infants in the Closer condition looked longer during familiarization than did infants in the Opener condition, this difference is insufficient to account for the between-condition differences observed in attention to New Goal and New Path events during test. First, infants in the Closer condition did not look significantly longer to either the first three or the last three habituation events (*p*'s>.13), suggesting that infants' increased attention to Closer familiarization events did not, for instance, lead them to attend more to the Closer claw's subsequent action, which might have allowed them to process the grasping action more completely. In addition, there is no effect of attention during familiarization on infants' attention to New Goal versus New Path test events: adding attention during familiarization as a covariate in a repeated-measures analysis of attention to New Goal versus New Path test events reveals no significant effects, either across condition (F_1,38_ = .19, *p*>.66, η_p_^2^ = .01) or within the Closer or Opener conditions alone (Closer condition: F_1,18_ = 1.36, *p*>.25, η_p_^2^ = .07; Opener condition: F_1,18_ = .85, *p*>.36, η_p_^2^ = .05). Finally, the independent interaction with condition on infants' attention to New Goal versus New Path events remains significant with the addition of attention during familiarization as a covariate (F_1,37_ = 7.43, *p<.*05, η_p_^2^ = .17), as does the tendency for infants in the Closer condition alone to look longer at New Goal than at New Path events (Closer condition repeated-measures ANOVA with familiarization as a covariate: F_1,18_ = 4.81; *p*<.05, η_p_^2^ = .21). Indeed, effect sizes for the effects of interest increase when the attention covariate is included in the analysis. Overall, then, infants' increased attention to Closer versus Opener familiarization events does not account for the observed between-condition differences in attention to New Goal versus New Path events during test.

### Discussion

Six-month-olds' looking times suggest they attributed agency to an inanimate claw that had previously exerted a negative effect on an agent, but not to an inanimate claw that had previously exerted a positive effect on an agent. This pattern of results suggests that negative outcomes are a cue to agency in infancy, as has been previously demonstrated in adulthood. These results are consistent with the body of evidence suggesting that infants and children show some negativity biases (reviewed in [46]), and represent the first piece of evidence that infants may rely on *valence*, in particular *social valence* determined by blocking an attempted goal, into their determination of whether or not an individual is an agent.

Yet, the observed pattern of results is also consistent with another hypothesis. Specifically, rather than evaluating the Protagonist's failed goal as negative, infants may have relied on some physical aspect of the behaviors involved (e.g., closing a box, the noise when a box slams shut, etc.), which lead them to attribute agency to the Closer claw. Indeed, though individual infants' attention during familiarization events did not influence their performance during test, as a group infants did attend longer to events that involved closing/slamming in Experiment 1. Thus, strong evidence for a negative agency bias requires demonstrating that infants truly evaluate the event as *socially negative*: while closing a box is not inherently bad, closing a box that an agent wishes to open is a negative, antisocial act, because it causes the agent to fail to achieve his or her goal.

To address this alternative explanation for the findings in Experiment 1, new groups of infants in Experiment 2 viewed a claw perform identical box-Opener (Opener condition) or box-Closer (Closer condition) actions as in Experiment 1; however, the actions were directed toward a non-agent (a third mechanical claw). At the start of each event, the non-agent claw engaged in box-directed actions like the puppet agent in Experiment 1 had: the non-agent claw turned to “face” the toy inside the box, it repeatedly lifted and dropped the box lid, etc. In addition, the end-states of the Opener and Closer familiarization events were physically the same as in Experiment 1: either the box was open and the non-agent claw contacted the toy, or the box was closed and the non-agent claw rested next to the box. Despite these similarities, we hypothesized that infants in Experiment 2 would not attribute a failed attempt to this third claw (see [63]), and therefore would not view the Opener/Closer claws' acts as leading to a positive or a negative outcome. Thus, if the results from Experiment 1 reflect a negative agency bias in particular, then infants should not attribute agency to any claw in Experiment 2 as neither causes a negative outcome.

## Experiment 2

### Methods

#### Participants

Participants were 40 6-month-olds (20 males; mean = 6;1; range: 5;17–6;15), of which 20 were randomly assigned to the Closer condition (9 females; range: 5;17–6;15) and 20 to the Opener condition (11 females; range: 5;17–6;15). Eight additional infants were run but excluded due to fussiness (3 in Opener condition, 2 in Closer condition) and experimenter error (2 in Opener condition, 1 in Closer condition). Exclusion rates were marginally higher in Experiment 1 than in Experiment 2 (Pearson's χ^2^ = 3.39; *p* = .07), in particular there was marginally fewer exclusions due to fussiness in Experiment 2 (Pearson's χ^2^ = 2.92; *p* = .09). We hypothesize that is due to the first half of participants in Experiment 1 being run with an all black curtain, resulting in generally higher rates of fuss-outs across all lab studies. Following changing the curtain to a light green color, we observed considerably fewer dropouts across studies.

#### Disclosure on sampling procedure

As in Experiment 1, each condition of Experiment 2 originally contained 16 infants. Four additional infants were added to each condition in Experiment 2 to equate sample sizes across Experiments.

#### Materials and Procedure

All procedures were identical to Experiment 1, except that during familiarization events, the Opener and Closer claws acted on a third claw covered in light brown duct tape (Figure 1C/D). A second independent coder, blind to condition, re-coded a random 25% of subjects' test events; the two coders reached 97% agreement. Additionally, we calculated the difference score between the original coder and the independent coder on each trial and computed the number of times that difference was in the hypothesized direction. This occurred on 28 out of the 60 recoded test trials.

### Results

#### Attention to Familiarization and Habituation events

Unlike in Experiment 1, there was no effect of condition on attention during familiarization, the first three habituation events, or the last three habituation events (repeated-measures ANOVA with attention to familiarization, the first 3 habituation events, and last 3 habituation events as within-subjects factors and condition as a between-subjects factor; F_2,76_ = .06, *p*>.93, η_p_^2^ = .002). Across condition infants looked equally to Opener and Closer familiarization events (average fam_Opener_ = 5.91 s (SEM = 1.41), average fam_Closer_ = 5.23 s (SEM = .68); F_1,38_ = .20, *p*>.65, η_p_^2^ = .005), equally to the first 3 grasping habituation events (first3hab_Closer_ = 6.48 s (.56); first3hab_Opener_ = 7.45 s (1.76); F_1,38_ = .28, *p*>.59; η_p_^2^ = .007), and equally to the last 3 grasping habituation events (last3hab_Closer_ = 2.78 s (.24); last3hab_Opener_ = 3.31 s (.55); F_1,38_ = .80, *p*>.37; η_p_^2^ = .02). Rate of habituation was also equivalent across condition: infants in the Opener condition habituated in an average of 9.9 trials (SEM = .50; 5 of 20 infants failed to habituate in 14 trials); infants in the Closer condition habituated in 8.3 trials (SEM = .51; 4 of 20 did not habituate; F_1,38_ = 2.68, *p*>.10, η_p_^2^ = .07).

#### Attention to Test events

**Figure 2:**
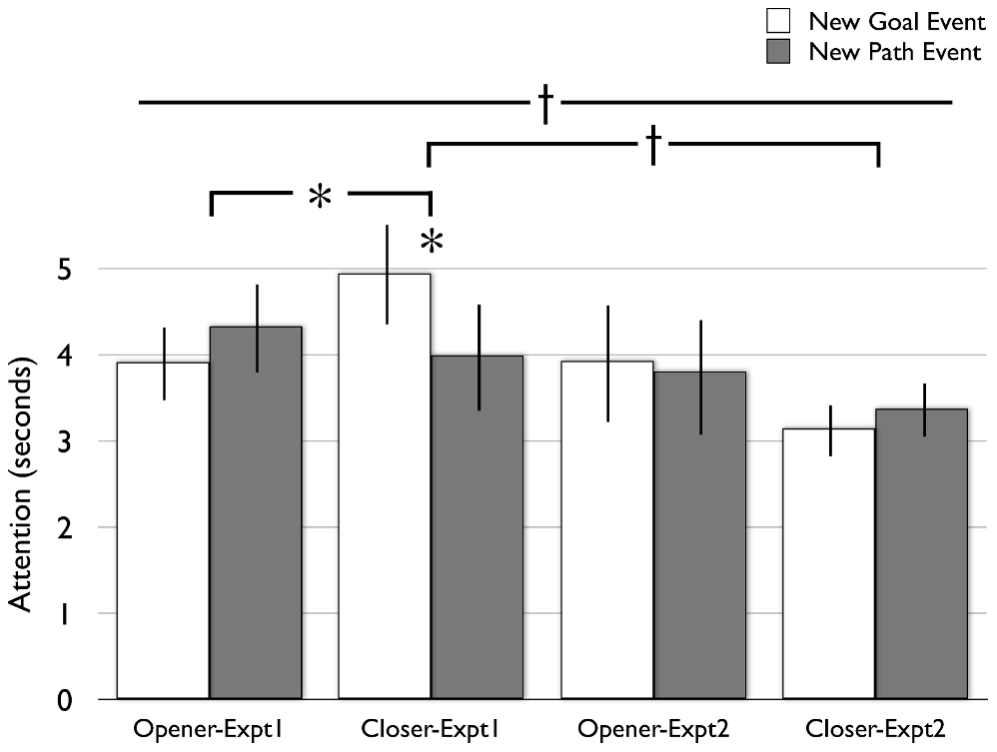
Looking time results. Infants' average attention during the 2 Familiarization events, the first three and the last three Habituation events, and the three New Goal and three New Path test events.

See Figure 2. As in Experiment 1, there were no condition differences in infants' overall attention during test events in Experiment 2 (AverageTestAttention_Closer_ = 3.24 s (.72), AverageTestAttention_Opener_ = 3.89 s (.87), F_1,38_ = 1.08, *p*>.30, η_p_^2^ = .03). In addition, a preliminary OMNIBUS ANOVA revealed no effect of age, sex, claw color, claw side during familiarization, attention during familiarization, targeted toy (ball or bear) during habituation, targeted toy side during habituation, attention to the first three or the last three habituation events, number of habituation events, whether or not the infant habituated in 14 events, or order of New Goal/Path events during test on infants' attention to New Goal versus New Path test events; subsequent analyses are collapsed across these variables.

We performed a repeated-measures ANOVA on infants' attention to New Goal and New Path test events as in Experiment 1, with condition as a between-subjects factor. This analysis revealed no main effect of infants' attention to New Goal versus New Path events (F_1,38_ = .01, *p*>.91, η_p_^2^<.0005) and no interaction with condition (F_1,38_ = .22, *p*>.64, η_p_^2^ = .006). Planned contrasts confirmed that infants failed to dishabituate to New Goal or New Path events in either the Opener or Closer conditions (last3hab_Opener_ = 3.31 s (.55), NewGoalTest_Opener_ = 3.93 s (.68), paired-t_19_ = −1.11, *p*>.28, η^2^ = .06; NewPathTest_Opener_ = 3.78 s (.66), paired-t_19_ = −.58; *p*>.59, η^2^ = .02; last3hab_Closer_ = 2.77 s (SEM = .24), NewGoalTest_Closer_ = 3.14 s (.29), paired-t_19_ = −1.33, *p*>.19, η^2^ = .09; NewPathTest_Closer_ = 3.39 s, paired-t_19_ = −1.44, *p*>.16, η^2^ = .09), and did not distinguish New Goal from New Path events in either condition (NewGoalTest_Opener_ = 3.93 s (.68), NewPathTest_Opener_ = 3.78 s (.66), paired-t_19_ = .21, *p*>.83, η^2^ = .002; NewGoalTest_Closer_ = 3.14 s (.29), NewPathTest_Closer_ = 3.39 s (.32), paired-t_19_ = −.58, *p*>.57, η^2^ = .02). As in Experiment 1, we examined individual infants' tendency to look longer to New Goal events than to New Path events during test: 11 of 20 infants in the Closer condition looked longer to New Goal than to New Path events (binomial *p*>.82), and 9 of 20 infants in the Opener condition did so (binomial p>.82; Pearson's χ^2^ = .4, *p*>.52).

## Cross-experiment comparisons

Every infant in Experiments 1 and 2 viewed familiarization events involving a claw that either opened or closed a box, and habituation and test events involving a claw reaching for a ball and a bear. Therefore, it is possible to compare infants' patterns of attention across Experiments.

### Attention to Familiarization/Habituation events

A repeated-measures ANOVA with attention during familiarization, the first three and the last three habituation events with Experiment (1 or 2) and condition (Opener or Closer) as between-subjects factors revealed no significant interactions (with Experiment: F_2,152_ = .65, *p*>.52, η_p_^2^ = .008; with Condition: F_2,152_ = 1.74, *p*>.17, η_p_^2^ = .02; with Experiment and Condition: F_2,152_ = .2.17, *p*>.11, η_p_^2^ = .03). In addition, rate of habituation did not differ across Experiment or condition: a univariate ANOVA comparing the number of events it took to reach the habituation criterion with Experiment and Condition as between-subjects factors revealed no significant effects or interactions (all p's>.19). Subsequent analyses were collapsed across attentional variables.

### Attention to Test events

A univariate ANOVA to infants' average attention during all test events (that is, not divided by New Goal and New Path events) with Condition and Experiment as between-subjects factors revealed no main effects and no interaction (Experiment: F_1,76_ = 2.33; *p*>.13, η_p_^2^ = .02; Condition: F_1,76_ = .09; *p*>.76, η_p_^2^ = .001; Interaction: F_1,76_ = 1.18; *p*>.28, η_p_^2^ = .02). That is, in addition to not differing by Condition within Experiments 1 and 2 as reported previously, infants did not look longer during test events as a whole within or across Conditions across Experiments 1 and 2.

A repeated-measures ANOVA comparing infants' attention to New Goal versus New Path events during test with Experiment and Condition as between-subjects factors revealed a marginally-significant three-way interaction with Experiment and Condition (F_1,76_ = 2.90, *p* = .09, η_p_^2^ = .04), but no main effect and no interaction with either Experiment alone or Condition alone, reflecting that it was only in the Closer condition in Experiment 1 that infants distinguished New Goal from New Path events. Follow-up analyses in which infants were grouped by whether they saw Opener or Closer familiarization events revealed a marginal interaction with Experiment in the Closer group (F_1,38_ = 3.84, *p* = .057, η_p_^2^ = .09), such that infants in the Closer group of Experiment 1 were more likely to distinguish New Goal from New Path events than were infants in the Closer group of Experiment 2; no such differences were observed in Opener groups across Experiments (F_1,38_ = .46, *p*>.50, η_p_^2^ = .01). Finally, individual infants' tendency to look longer to New Goal versus New Path events in across all conditions revealed a similar marginally-significant interaction (Pearson χ^2^ (3) = 6.65, *p* = .08); this interaction is present when comparing the Closer conditions only (Pearson χ^2^ (1) = 2.85, *p* = .09), but not when comparing the Opener conditions only (Pearson χ^2^ (1) = 0, *p* = 1). Though these cross-experiment interactions are all marginal, they generally support the significant findings from Experiment 1: only those infants who viewed a claw *cause a negative outcome* subsequently attended to the claw's object-directed action as though they had attributed agency to it, looking longer when the claw “changed its mind” than when the claw changed its path of motion; this pattern of results was observed using both parametric and non-parametric tests.

## General Discussion

The data reported here add to a growing literature suggesting that human infants are highly attuned to the social world. Previous studies have shown that infants rapidly distinguish agents from non-agents [21], [23], [67], reason about agents' goal-directed behaviors [24], [37], [68], evaluate the actions of agents based specifically on their prosocial and antisocial nature [63], [69], [71], and even privilege the intentional content of prosocial and antisocial acts over the specific outcomes those acts are associated with [72], [73]. The current studies provide evidence that for infants, as for adults, not only do judgments of agency influence social evaluations, but social evaluations influence judgments of agency.

Across two experiments, six-month-olds who observed a mechanical claw inflict a negative outcome (blocking an agent's goal) subsequently attributed agency to that claw, whereas infants who observed a claw inflict a positive outcome (facilitating a goal), or who saw a claw carry out physically identical but non-valenced actions (opening or closing a box) did not. Such findings are consistent with recent work with adults demonstrating that while neutral, everyday events are regularly attributed to physical forces or random chance by adult observers, excessively negative outcomes tend to be attributed to malevolent external agents [41]. Adding to previous developmental evidence for a general “negativity bias” in which negative social agents are privileged in infants' and children's memory, learning processes, and evaluations (see [46] for a review; see also [50]–[52]). In the current studies infants used negative social outcomes to determine whether a particular causal entity is or is not an agent in the first place. These results suggest that infants' agency-representations involve more than just the physical and spatiotemporal properties of an object and its actions, and include an analysis of its social-relational interactions (see also [74]).

Evidence for a negative agency bias in both adults and 6-month-old infants raises questions about the role of experience in its emergence. Specifically, while it seems unlikely that infants' tendency to attribute agency to the causes of negative outcomes is due to motivated reasoning or a desire to “save face” as is often suggested as a reason in adult research [54]–[59], perhaps infants' bias is the result of rapidly-acquired associations between outcome valence and the likely presence of agents in their daily lives. While possible, on further investigation it seems that if anything, infants' experiences should encourage the development of a *positive* agency bias, rather than a negative one as shown here. Indeed, the great majority of infants' daily experiences come through interactions with adult caregivers, whose primary responsibility is to meet the needs of their relatively helpless children (changing dirty diapers, providing sustenance and physical protection, lending social and emotional support, etc.). These interactions presumably increase positive and decrease negative experiences, and should encourage the development of an association between agents and positive outcomes, not negative ones.

Recent work by Newman et al. [30], demonstrating that by 12 months of age infants selectively associate agency with ordered stimuli, may be consistent with an experience-driven account of the development of agency representations. That is, 12-month-olds (but not 7-month-olds) look longer at events in which physical order (for example, neatly stacked blocks) seems to have been created by a non-agent versus an agent, suggesting they see agents as uniquely capable of creating order. Underlying this effect may be that 12-month-olds have had routine opportunity to see agents creating order in their daily lives, leading them to associate agents and order, but few or no opportunities to see non-agents creating order. In contrast, infants look equally to events in which agents and non-agents create disorder; this is presumably also consistent with their daily experiences. Although infants in the current studies are significantly younger than 12 months, and though “ordered” and “positive” are not synonymous, it has recently been demonstrated that both infants and preschool children view ordered objects to be a positive stimulus and disordered objects to be an aversive stimulus [75], suggesting the concepts may be connected from early in life. Although the exact nature of the relationship between positivity/negativity and order/disorder in infants' agency representations remains to be elucidated, both previous work and an analysis of infants' likely daily experiences suggest that if anything, infants should tend to ascribe agency to the causes of *positive* outcomes, not negative ones as seen here, and speak against an experiential account of the current results.

Several unanswered questions remain. First, future studies should examine whether, given clearly agentive causes of *both* negative and positive social outcomes (that is, when all entities are animate and no claws are involved) infants would ascribe relatively *more* goal-directedness (more agency) to agents that caused negative versus positive outcomes, just as adults and children ascribe more intentionality to agentic actions that bring about bad versus good side effects (e.g., [39], [42]). Although it is rather difficult to imagine an infant methodology that allows for measuring *how much* agency infants ascribe to an entity, there is recent evidence that meaningful information can be gleaned from infants' relative surprise to particular outcomes [76], perhaps a similar methodology could be utilized here. In addition, from the current studies it is unclear whether infants *never* attribute agency to inanimate entities that cause positively-valenced outcomes, or whether the act of opening a box was just not sufficiently positive for them to do so (or whether infants attributed a level of agency to the Opener claw that was insufficient to guide specific goal-attribution in the Woodward task). While adults tend to attribute agency to the causes of negative outcomes more easily, and more often, than to the causes of positive outcomes, there is some evidence that particularly positive outcomes may lead to agency attributions as well (e.g., [8]). It is up to future studies to elucidate whether the asymmetry in agency attribution viewed here is present for other instances of positive and negative social outcomes in infancy, and/or whether there are any positive outcomes that do lead infants to attribute agency (enough to support specific goal-attribution as in the Woodward task) to non-agentive causes.

Finally, this work speaks more generally to the question of the flexibility/malleability of infants' initial determination of an entity's status as an agent or a non-agent. That is, after learning whether that object was associated with an outcome of a particular type or valence, can infants shift their assessments from non-agent to agent and vise versa? Whether infants can modify their initial agency attributions is an important question, as it bears on the flexibility of infant's object and agent concepts and their ability to update existing representations with new information in a dynamic fashion. Unfortunately, previous findings relevant to this question are ambiguous. For instance, in Newman et al. 's [30] Experiment 3, infants were *habituated* to a non-agent creating order, to determine whether infants could learn that a particular non-agent can create order, despite whatever assumptions they typically hold. Despite this repeated experience, however, infants were still relatively more surprised by the non-agent creating order (a scene they were now very familiar with) than they were by an unfamiliar agent doing so (an unfamiliar scene). These results suggest that infants' agency-attributions are fairly rigid, and unlikely to be updated based on seeing a non-agent performing agent-like behavior. In contrast, work by Johnson and colleagues [34], [73], also with 12-month-olds, has shown that infants who view a typical non-agent engage in contingent interaction with a known agent *will* attribute agency to that non-agent in the future (as measured by their readiness to follow its “gaze”, and by the Woodward paradigm as in the current studies). That is, Johnson and colleagues' results suggest that infants' agency-attributions are fairly fluid, and updatable with new information. Clearly, further study is required to disentangle these apparently conflicting results, and to elucidate the exact computational processes involved in infants' and adults' construction, and adjustment, of agent-representations based on various inputs. The current studies represent an important piece of evidence from which to build, supporting the idea that agency-representations are fluid and updatable from very early in life.

## References

[1] Baron-Cohen S (1995) Mindblindness: An essay on autism and theory of mind. Cambridge, MA: MIT Press.

[2] Klin A (2000) Attributing social meaning to ambiguous social stimuli in higher functioning autism and Asperger syndrome: The social attribution task. Journal of Child Psychology and Psychiatry 41: 831–846. 11079426

[3] Rutherford MD, Pennington BF, Rogers SJ (2006) The perception of animacy in young children with autism. Journal of Autism and Developmental Disorders 36: 983–992. 16897392 10.1007/s10803-006-0136-8

[4] Haselton MG, Buss DM (2000) Error management theory: A new perspective on biases in cross-sex mind reading. Journal of Personality and Social Psychology 78: 81–91. 10653507 10.1037//0022-3514.78.1.81

[5] Nesse RM (2001) The smoke detector principle. Natural selection and the regulation of defensive responses. Ann N Y Academy of Science 935: 75–85. 11411177

[6] Barrett JL (2000) Exploring the natural foundations of religion. Trends in Cognitive Sciences 4: 29–34. 10637620 10.1016/s1364-6613(99)01419-9

[7] Boyer P (2001) Religion explained: The evolutionary origins of religious thought. New York, NY: Basic Books.

[8] Gilbert DT, Brown RP, Pinel EC, Wilson TD (2000) The illusion of external agency. Journal of Personality and Social Psychology 79: 690–700. 11079235 10.1037//0022-3514.79.5.690

[9] Guthrie S (1993) Faces in the clouds. New York, NY: Oxford University Press.

[10] Heider F, Simmel M (1944) An experimental study of apparent behavior. The American Journal of Psychology 57: 243–259.

[11] Hume D (1757/1957) The natural history of religion. Stanford, CA: Stanford University Press.

[12] Piaget J (1929) The child's conception of the world. London: Routledge & Kegan Paul.

[13] Camilleri JA, Kuhlmeier VA, Chu JYY (2010) Remembering helpers and hinderers depends on behavioral intentions of the agent and psychopathic characteristics of the observer. Evolutionary Psychology 8: 303–316. 22947799 10.1177/147470491000800213 PMC10481016

[14] Rossett E (2008) It's no accident: Our bias for intentional explanations. Cognition 108: 771–780. 18692779 10.1016/j.cognition.2008.07.001

[15] Baron-Cohen S (1994) The Mindreading System: new directions for research. Current Psychology of Cognition 13: 724–750.

[16] Biro S, Leslie AM (2007) Infants' perception of goal-directed actions: Development through cue-based bootstrapping. Developmental Science 10: 379–398. 17444978 10.1111/j.1467-7687.2006.00544.x

[17] Carey S, Spelke E (1994) Domain-specific knowledge and conceptual change. In:Hirschfeld LA, Gelman SA, editors. Mapping the mind: domain specificity in cognition and culture. New York, NY Cambridge University Press. 169–200.

[18] Gergely G, Cisbra G (2003) Teleological reasoning in infancy: The naïve theory of rational action. Trends in Cognitive Sciences 7: 287–292. 12860186 10.1016/s1364-6613(03)00128-1

[19] Johnson S, Slaughter V, Carey S (1998) Whose gaze will infants follow? The elicitation of gaze-following in 12 -month-olds. Developmental Science 1: 233–238.

[20] Kuhlmeier V, Wynn K, Bloom P (2003) Attribution of dispositional states by 12- month-olds. Psychological Science 14: 402–408. 12930468 10.1111/1467-9280.01454

[21] Legerstee M (1992) A review of the animate-inanimate distinction in infancy: implications for models of social and cognitive knowing. Early Development and Parenting 1: 59–67.

[22] Legerstee M, Markova G (2008) Variations in 10-month-old infant imitation of people and things. Infant Behavior and Development 31: 81–91. 17719646 10.1016/j.infbeh.2007.07.006

[23] Leslie AM (1994) ToMM, ToBy, and Agency: Core architecture and domain specificity. In: Hirschfeld L, Gelman S, editors. Mapping the mind: Domain specificity in cognition and culture New York, NY Cambridge University Press. 119–148.

[24] Luo Y, Baillargeon R (2005) Can a self-propelled box have a goal?: Psychological reasoning in 5-month-old infants. Psychological Science 16: 601–608. 16102062 10.1111/j.1467-9280.2005.01582.x PMC3351378

[25] Luo Y (2011) Three-month-old infants attribute goals to a non-human agent. Developmental Science 14: 453–460. 22213913 10.1111/j.1467-7687.2010.00995.x

[26] Meltzoff A (1995) Understanding the intentions of others: Re-enactment of intended acts by 18-month-old children. Developmental Psychology 31: 838–850. 25147406 10.1037/0012-1649.31.5.838 PMC4137788

[27] Meltzoff AN (2007) ‘Like me': A foundation for social cognition. Developmental Science 10: 126–134. 17181710 10.1111/j.1467-7687.2007.00574.x PMC1852489

[28] Meltzoff AN, Moore MK (1994) Imitation, memory, and the representation of persons. Infant Behavior and Development 17: 83–99. 25147416 10.1016/0163-6383(94)90024-8 PMC4137868

[29] Muentener P, Carey S (2010) Infants' causal representations of state change events. Cognitive Psychology 61: 63–86. 20553762 10.1016/j.cogpsych.2010.02.001 PMC2930082

[30] Newman GE, Keil FC, Kuhlmeier V, Wynn K (2010) Sensitivity to design: Early understandings of the link between agents and order. Proceedings of the National Academy of Sciences 107: 17140–17145. 10.1073/pnas.0914056107 PMC2951444 20855603

[31] Premack D (1990) The infant's theory of self-propelled objects. Cognition 36: 1–16. 2383967 10.1016/0010-0277(90)90051-k

[32] Rakison D, Poulin-Dubois D (2001) The developmental origin of the animate- inanimate distinction. Psychological Bulletin 2: 209–228. 10.1037/0033-2909.127.2.209 11316011

[33] Ruffman T, Taumoepeau M, Perkins C (2012) Statistical learning as a basis for social understanding in children. British Journal of Developmental Psychology 30: 87–104. 22429035 10.1111/j.2044-835X.2011.02045.x

[34] Shimizu YA, Johnson SC (2004) Infants' attribution of a goal to a morphologically unfamiliar agent. Developmental Science 7: 425–430. 15484590 10.1111/j.1467-7687.2004.00362.x

[35] Sommerville JA, Woodward AL, Needham A (2005) Action experience alters 3-month-old infants' perception of others' actions. Cognition 96: B1–B11. 15833301 10.1016/j.cognition.2004.07.004 PMC3908452

[36] Tomasello M, Carpenter M, Call J, Behne T, Moll H (2005) In search of the uniquely human. Behavioral and Brain Science 28: 721–727. 10.1017/S0140525X05000129 16262930

[37] Woodward A (1998) Infants selectively encode the goal of an actor's reach. Cognition 69: 1–34. 9871370 10.1016/s0010-0277(98)00058-4

[38] Csibra G (2008) Goal attribution to inanimate agents by 6.5-month old infants. Cognition 107: 705–717. 17869235 10.1016/j.cognition.2007.08.001

[39] Knobe J (2003) Intentional action in folk psychology: An experimental investigation. Philosophical Psychology 16: 309–324.

[40] Knobe J (2005) Theory of mind and moral cognition: Exploring the connections. Trends in Cognitive Sciences 9: 357–359. 16006176 10.1016/j.tics.2005.06.011

[41] Morewedge CK (2009) Negativity bias in attribution of external agency. Journal of Experimental Psychology: General 138: 535–545. 19883135 10.1037/a0016796

[42] Leslie A, Knobe J, Cohen A (2006) Acting intentionally and the side-effect effect: Theory of mind and moral judgment. Psychological Science 17: 421–427. 16683930 10.1111/j.1467-9280.2006.01722.x

[43] Luczak H, Roetting M, Schmidt L (2003) Let's talk: Anthropomorphization as a means to cope with stress of interacting with technical devices. Ergonomics 46: 1361–1374. 14612325 10.1080/00140130310001610883

[44] Waytz A, Morewedge CK, Epley N, Montelone G, Gao JH, et al (2010) Making sense by making sentient: Effectance motivation increases anthropomorphism. Journal of Personality and Social Psychology 99: 410–435. 20649365 10.1037/a0020240

[45] Ward AF, Olsen A, Wegner DM (2013) The harm-made mind: Observing victimization augments attribution of minds to vegetative patients, robots, and the dead. Psychological Science 24: 1427–1435. 10.1177/0956797612472343 23749051

[46] Vaish A, Grossmann T, Woodward A (2008) Not all emotions are created equal: The negativity bias in social-emotional development. Psychological Bulletin 13: 383–403. 10.1037/0033-2909.134.3.383 PMC3652533 18444702

[47] Baumeister RF, Bratslavsky E, Finkenauer C, Vohs KD (2001) Bad is stronger than good. Review of General Psychology 5: 323–370.

[48] Kanouse DE, Hanson LR (1971) Negativity in evaluations. In: Jones EE, Kanouse DE, Kelley HH, Nisbett RE, Valins S, Weiner B, editors. Attribution: Perceiving the causes of behavior. Morristown, NJ: General Learning Press. pp. 47–62.

[49] Rozin P, Royzman EB (2001) Negativity bias, negativity dominance, and contagion. Personality and Social Psychology Review 5: 296–320.

[50] Kinzler DK, Shutts K (2008) Memory for “mean” over “nice”: The influence of threat on children's face memory. Cognition 107: 775–783. 18001702 10.1016/j.cognition.2007.09.005 PMC2390832

[51] Hamlin JK, Wynn K (2012) Who knows what's good to eat? Infants fail to match the food preferences of antisocial others. Cognitive Development 27: 227–239.

[52] Hamlin JK, Wynn K, Bloom P (2010) Three-month-old infants show a negativity bias in social evaluation. Developmental Science 13: 923–929. 20977563 10.1111/j.1467-7687.2010.00951.x PMC2966030

[53] Barrett JL (2004) Why would anyone believe in God? Walnut Creek, CA: AltaMira Press.

[54] Bar-Anan Y, Wilson TD, Gilbert DT (2009) The feeling of uncertainty intensifies affective reactions. Emotion 9: 123–127. 19186925 10.1037/a0014607

[55] Epley N, Waytz A, Akalis S, Cacioppo JT (2008) When we need a human: Motivational determinants of anthropomorphism. Social Cognition 26: 143–155.

[56] Jones EE, Davis KE (1965) From acts to dispositions: the attribution proces in social psychology. In: Berkowitz L, editor. Advances in experimental social psychology, Vol II. New York: Academic Press. 219–266.

[57] Norenzayan A, Hansen IG (2006) Belief in supernatural agents in the face of death. Personality and Social Psychology Bulletin 32: 174–187. 16382080 10.1177/0146167205280251

[58] Taylor SE (1991) The asymmetrical impact of positive and negative events: The mobilization-minimization hypothesis. Psychological Bulletin 110: 67–85. 1891519 10.1037/0033-2909.110.1.67

[59] Taylor SE, Brown JD (1988) Illusion and well-being: A social psychological perspective on mental health. Psychological Bulletin 103: 193–210. 3283814

[60] Ward AF, Olsen A, Wegner DM (2013) The harm-made mind: Observing victimization augments attribution of minds to vegetative patients, robots, and the dead. Psychological Science 24: 1427–1435. 10.1177/0956797612472343 23749051

[61] Premack D, Premack A (1997) Infants attribute value± to the goal-directed actions of self-propelled objects. Journal Of Cognitive Neuroscience 9: 848–856. 23964604 10.1162/jocn.1997.9.6.848

[62] Leslie A, Knobe J, Cohen A (2006) Acting intentionally and the side-effect effect: Theory of mind and moral judgment. Psychological Science 17: 421–427. 16683930 10.1111/j.1467-9280.2006.01722.x

[63] Hamlin JK, Wynn K (2011) Young infants prefer prosocial to antisocial others. Cognitive Development 26: 30–39. 21499550 10.1016/j.cogdev.2010.09.001 PMC3076932

[64] Hamlin JK, Wynn K, Bloom P (2007) Social evaluation by preverbal infants. Nature 450: 557–559. 18033298 10.1038/nature06288

[65] Hamlin JK, Newman G, Wynn K (2009) 8-month-olds infer unfulfilled goals, despite contrary physical evidence. Infancy 14: 579–590. 10.1080/15250000903144215 32693534

[66] Johnson SC, Booth A, O'Hearn K (2001) Inferring the goals of a nonhuman agent. Cognitive Development 16: 637–656.

[67] Morton J, Johnson MM (1991) CONSPEC and CONLERN: a two-process theory of infant face recognition. Psychological Review 98: 164–181. 2047512 10.1037/0033-295x.98.2.164

[68] Gergely G, Nádasdy Z, Csibra G, Bíró S (1995) Taking the intentional stance at 12 months of age. Cognition 56: 165–193. 7554793 10.1016/0010-0277(95)00661-h

[69] Geraci A, Surian L (2011) The developmental roots of fairness: Infants' reactions to equal and unequal distributions of resources. Developmental Science 14: 1012–1020. 21884317 10.1111/j.1467-7687.2011.01048.x

[70] Casstevens RM (2007). jHab: Java habituation software (version 1.0.2) [computer software]. Chevy Chase, MD.

[71] Hamlin JK (2013a) Moral judgment and action in preverbal infants and toddlers: Evidence for an innate moral core. Current Directions in Psychological Science 22: 186.

[72] Hamlin JK (2013b) Failed attempts to help and harm: Intention versus outcome in preverbal infants' social evaluations. Cognition 128: 451–474. 23811094 10.1016/j.cognition.2013.04.004

[73] Hamlin JK, Ullman T, Tenenbaum J, Goodman N, Baker C (2013) The mentalistic basis of core social cognition: Experiments in preverbal infants and a computational model. Developmental Science 16: 209–226. 23432831 10.1111/desc.12017 PMC4100482

[74] Johnson SC, Slaughter V, Carey S (1998) Whose gaze will infants follow? The elicitation of gaze-following in 12-month-olds. Developmental Science 1: 233–238.

[75] Pun A, Baron AS (2013). Distinguishing positive and negative intergroup attitudes in infancy. Poster presented at the Biennial Meeting of the Society for Research in Child Development.

[76] Teglas E, Vul E, Girotto V, Gonzalez M, Tenebaum JB, et al (2011) Pure reasoning in 12-month-old infants as probabilistic inference. Science 332: 1054–1059. 21617069 10.1126/science.1196404

